# Sequential Sampling and Estimation of Approximately Bandlimited Graph Signals

**DOI:** 10.3390/s21041460

**Published:** 2021-02-19

**Authors:** Sijie Lin, Ke Xu, Hui Feng, Bo Hu

**Affiliations:** 1 Research Center of Smart Networks and Systems, School of Information Science and Technology, Fudan University, Shanghai 200433, China; sjlin18@fudan.edu.cn (S.L.); bohu@fudan.edu.cn (B.H.); 2 Center for Intelligent Medical Electronics, School of Information Science and Technology, Fudan University, Shanghai 200433, China; kexu18@fudan.edu.cn

**Keywords:** graph signal, sequential sampling, consistent estimation

## Abstract

Graph signal sampling has been widely studied in recent years, but the accurate signal models required by most of the existing sampling methods are usually unavailable prior to any observations made in a practical environment. In this paper, a sequential sampling and estimation algorithm is proposed for approximately bandlimited graph signals, in the absence of prior knowledge concerning signal properties. We approach the problem from a Bayesian perspective in which we formulate the signal prior by a multivariate Gaussian distribution with unknown hyperparameters. To overcome the interconnected problems associated with the parameter estimation, in the proposed algorithm, hyperparameter estimation and sample selection are performed in an alternating way. At each step, the unknown hyperparameters are updated by an expectation maximization procedure based on historical observations, and then the next node in the sampling operation is chosen by uncertainty sampling with the latest hyperparameters. We prove that under some specific conditions, signal estimation in the proposed algorithm is consistent. Subsequent validation of the approach through simulations shows that the proposed procedure yields performances which are significantly better than existing state-of-the-art approaches notwithstanding the additional attribute of robustness in the presence of a broad range of signal attributes.

## 1. Introduction

Graph signal is a powerful tool to represent and analyze data with irregular interconnections by defining signal values on graph vertices and assigning edge weights according to correlation or similarity [1]. In the past decade, graph signal processing theory has developed rapidly, extending classical signal processing techniques such as Fourier analysis [1,2], filtering [3,4,5], sampling and interpolation [6,7,8] to graph signal setting. Related concepts and methods have found wide application in sensor networks [9,10], brain analysis [11], image processing [12], three-dimensional (3D) point cloud processing [13], and machine learning [14,15,16].

Sampling for (lossless) reconstruction or (minimum error) estimation is a fundamental problem in graph signal processing. For example, a sample survey in a social network needs to carefully select interviewees so as to better predict the attitudes of all users. In a sensor network, it is important to optimize sensor placement so that more information can be collected by a restricted number of sensors due to economic constraint [9]. In addition, any graph-based active semisupervised learning task may also be interpreted as a graph signal sampling and estimation problem [14,15].

There have been multiple works on graph signal sampling. The authors of [6,7] try to extend the Nyquist sampling theory to bandlimited graph signals. More works are seeking optimal sampling sets for graph signal estimation, where the graph signals are usually assumed to be bandlimited [17,18,19] or approximately bandlimited [17,20] in the frequency domain, or smooth in the vertex domain [17,21]. Sampling strategies mainly include topology-based methods that compute a score for each vertex [18,21], and design-of-experiment (DOE) approaches that optimize certain scalarization form of some target matrix [19,22]. Most of the current sampling methods rely on accurate bandwidth [6,18,19] or prior distribution [22,23] of the graph signal.

It is natural in many applications to assume that the graph signal is somehow bandlimited or smooth. For instance, people with strong social connection are likely to hold similar opinions and temperature sensors located close to each other usually get similar measurements. However, in practice it is hard to know the exact bandwidth or prior distribution of the graph signal before any observation. In such cases, those sampling strategies based on accurate signal model become not applicable.

A possible solution is to sample and estimate in a sequential way. Unknown signal properties can be estimated from previous observations, and the subsequent sampling node can be selected under the latest signal model. Such a sequential framework can overcome the difficulty of incomplete system model and take advantage of model-based sample selection and signal estimation [20].

In this work, we investigate the sequential sampling and estimation of approximately bandlimited (ABL) graph signals, whose bandwidth and energy level are both not known in advance. With the aid of a Butterworth low-pass filter, the ABL graph signal is assumed to follow a multivariate Gaussian distribution with unknown hyperparameters. The variance of observation noise is also considered unknown.

In the proposed algorithm, hyperparameter estimation and sample selection are performed in an alternating way. At each step, expectation maximization (EM) [24] is first used to update the maximum marginal likelihood (MML) estimation of unknown hyperparameters based on historical observations. Substituting these latest estimated values into the signal prior and noise distribution, a Bayesian estimation and prediction procedure can then be performed. According to the uncertainty sampling (US) criterion [25], the node with the largest predictive variance is selected as the next node to sample.

In fact, estimating prior distribution from data, namely, empirical Bayes (EB) method, has long been studied [26]. It has been proved that under certain assumptions, the EB posterior distribution of parameter is consistent at its true value [27]. Meanwhile, an alternating approach analogous to the proposed one has been used in sequential DOE for nonlinear least squares (LS) regression. There, parameter estimation is proved to be consistent, and the design is asymptotically optimal [28].

In this paper, we prove that under specific conditions, signal estimation in the proposed algorithm is consistent at the true value. That is, as the number of samples goes to infinity, the estimated graph signal can get arbitrarily close to the true value. The finite-time performance of the proposed algorithm is validated by simulation results. Our algorithm is able to adjust to ABL graph signals with different properties, and it performs better in estimation error than existing methods.

The rest of the paper is organized as follows. We detail the signal prior and observation model in Section 2, and develop a sequential sampling and estimation algorithm in Section 3. The consistency of the proposed algorithm is proved under specific conditions in Section 4, and its finite-time performance is validated by experiments in Section 5. We conclude the paper in Section 6.

Throughout the paper, we use normal-weight italic lowercase letters (e.g., *x*) to denote scalar variables, bold italic lowercase letters (e.g., x) to denote vectors, bold italic uppercase letters (e.g., X) to denote matrices, and calligraphic uppercase letters (e.g., X) to denote sets.

## 2. System Model

### 2.1. Preliminaries

We consider a simple connected weighted undirected graph G=(V,E,W), where V is the set of vertices indexed by {1,…,N}; E is the set of edges between vertex pairs, e.g., (i,j)∈E denotes an edge between vertex *i* and vertex *j*; and W is the weighted adjacency matrix, with $w_{ij}$ the weight of edge between vertex *i* and vertex *j* if they are connected, or 0 otherwise. A graph signal that takes a real value on each vertex of the graph can be represented as $f\in R^{N}$, where $f_{i}$ is the signal value on vertex *i*.

The Laplacian matrix is defined as L=D−W, where $D=\ldots (d_{1},\ldots ,d_{N})$ with $d_{i}=\sum_{j:(i,j)\in E}w_{ij}$ is the degree matrix. Being symmetric and positive semi-definite, L has a spectral decomposition $L=V\Lambda V^{T}$, where Λ is a diagonal matrix of the eigenvalues $0=\lambda_{1}<\lambda_{2}\leq \cdots \leq \lambda_{N}$, and V contains the corresponding orthonormal eigenvectors ${\left\{v_{k}\right\}}_{k=1}^{N}$ as columns. These eigenvectors act as graph Fourier basis, and the associated eigenvalues are regarded as graph frequencies [1]. The frequency-domain coefficients $\hat{f}$ of a graph signal f can be calculated via the graph Fourier transform (GFT) as $\hat{f}=V^{T}f$. The graph signal f can be expressed as $f=V\hat{f}$, which is known as the inverse GFT (IGFT).

### 2.2. Signal Prior

In this work, the graph signal f is assumed to be drawn from the following distribution:

(1) $$p\left(f;\alpha ,\gamma \right)=N(f\;|\;0,{\left(\alpha I_{N}+\gamma L^{2n}\right)}^{-1}),$$

where $I_{N}$ denotes the N×N identity matrix, and n,α,γ>0 are hyperparameters, among which α,γ are assumed unknown in prior. The meanings of the signal model and hyperparameters are explained in the frequency domain as follows.

By GFT, the spectral coefficients ${\left\{{\hat{f}}_{k}\right\}}_{k=1}^{N}$ follow independent zero-mean Gaussian distributions with variances conforming to a Butterworth low-pass filter ([29], Section 7.3):

(2) $$p\left({\hat{f}}_{k}\right)=N\;\left({\hat{f}}_{k}\;|\;0,\frac{g^{2}}{1+{\left(\lambda_{k}/\lambda_{c}\right)}^{2n}}\right),$$

where *n* is the order, $\lambda_{c}=\sqrt[{2n}]{\alpha /\gamma }$ is the cut-off frequency, and $g=1/\sqrt{\alpha }$ is the amplitude gain of the filter. Under such a prior, we say the graph signal is *approximately bandlimited* in a probabilistic sense, where $\lambda_{c}$ plays the role of bandwidth, *n* controls the strictness of banlimitedness, and *g* determines the energy level of the graph signal.

Specially, when n=1/2 and δ=α/γ is small, the signal prior Equation (1) becomes

(3) $$p\left(f\right)\propto \exp\;\left(-\frac{\gamma }{2}f^{T}\left(L+\delta I_{N}\right)f\right),$$

which is widely used in graph signal sampling and estimation [9,23]. The restriction on total variation $f^{T}Lf=\sum_{(i,j)\in E}w_{ij}{\left(f_{i}-f_{j}\right)}^{2}$ can lead to smooth signals over the graph.

If n→∞, the out-of-band coefficients become deterministic zeros. The graph signal becomes strictly bandlimited, represented by

(4) $$f=V_{K}{\hat{f}}_{K},$$

where $K=\max\{k\;|\;\lambda_{k}\leq \lambda_{c}\}$, ${\hat{f}}_{K}$ collects the first *K* GFT coefficients that may be non-zero, and $V_{K}$ contains the first *K* GFT basis.

In conclusion, our signal prior Equation (1) is flexible to describe approximately bandlimited graph signals with different bandwidth, strictness of bandlimitedness, and energy level. It can also cover the most commonly used smoothness prior [9,23] and bandlimitedness prior [6,7,18,19]. Additionally, this model is simple with three hyperparameters, and can be represented in the vertex domain without eigendecomposition.

Here, hyperparameters α,γ or equivalently $\lambda_{c},g$ in the signal prior are assumed unknown, that is, we do not need to have prior knowledge about the bandwidth or energy level of the graph signal. Only a value for *n* is required to control the strictness of bandlimitedness of the estimated signal. Compared to those methods for only strictly bandlimited graph signals with known bandwidth or smooth graph signals with known prior distribution, our signal prior is more general.

### 2.3. Observation Model

To overcome the difficulty caused by unknown hyperparameters, a sequential sampling process is considered, where one node is sampled at each step, so that unknown hyperparameters can be estimated from previous observations, and the next node to sample can be selected using the latest hyperparameters.

Suppose that vertex $s_{t}$ is sampled at step *t*, and the observed signal value is $y_{t}$. The observation model can be expressed as

(5) $$y_{t}=\psi_{t}^{T}f+w_{t},$$

where $\psi_{t}^{T}=e_{s_{t}}^{T}$ is the sampling vector with its $s_{t}$-th element equal to 1 and others equal to 0, and $w_{t}$ is an additive zero-mean Gaussian observation noise with unknown precision β,

(6) $$p\left(w_{t};\beta \right)=N\left(w_{t}\;|\;0,\beta^{-1}\right).$$

In hyperparameter, signal estimation, and sample selection at step *t*, the historical observations from step 1 to step *t* are considered together. Let

(7) $$y_{1:t}=\left[\begin{array}{c} y_{1}\\ \vdots \\ y_{t} \end{array}\right],\;\Psi_{1:t}=\left[\begin{array}{c} \psi_{1}^{T}\\ \vdots \\ \psi_{t}^{T} \end{array}\right]=\left[\begin{array}{c} e_{s_{1}}^{T}\\ \vdots \\ e_{s_{t}}^{T} \end{array}\right],\;w_{1:t}=\left[\begin{array}{c} w_{1}\\ \vdots \\ w_{t} \end{array}\right].$$

The noise values $w_{1},\ldots ,w_{t}$ involved in different samples (on either different vertices or the same vertex) are assumed to be independent and identically distributed (i.i.d.). According to Equations (5) and (6),

(8) $$\begin{array}{c} y_{1:t}=\Psi_{1:t}f+w_{1:t}, \end{array}$$

(9) $$\begin{array}{c} p\left(w_{1:t};\beta \right)=N\left(w_{1:t}\;|\;0,\beta^{-1}I_{t}\right). \end{array}$$

### 2.4. Problem Formulation

Under the signal and observation model described in Section 2.2 and Section 2.3, at each step *t* in the sequential sampling and estimation process, the two core problems are (1) how to estimate the unknown hyperparameters α,γ,β as well as the graph signal f based on the historical observations $\Psi_{1:t},y_{1:t}$

(10) $$\alpha_{t}^{\prime },\gamma_{t}^{\prime },\beta_{t}^{\prime },f_{t}^{\prime }↤\Psi_{1:t},y_{1:t},$$

where $\alpha_{t}^{\prime },\gamma_{t}^{\prime },\beta_{t}^{\prime }$ are the estimated values of hyperparameters at step *t*, and $f_{t}^{\prime }$ denotes the estimated graph signal at step *t*, and (2) how to select the next node $s_{t+1}$ to be sampled at step t+1 with the latest estimated values of hyperparameters

(11) $$s_{t+1}↤\Psi_{1:t},y_{1:t},\alpha_{t}^{\prime },\gamma_{t}^{\prime },\beta_{t}^{\prime }.$$

Our ultimate goal is to minimize signal estimation error within given budget, or from another angle, reach certain estimation accuracy with least samples.

## 3. Algorithm

### 3.1. Hyperparameter and Signal Estimation

We first focus on unknown hyperparameter estimation from observation data. Here, maximum marginal likelihood (MML) estimation of unknown hyperparameters is adopted:

(12) $$\begin{array}{cc} \alpha_{t}^{\prime },\gamma_{t}^{\prime },\beta_{t}^{\prime } & =\arg\max_{\alpha ,\gamma ,\beta }p\left(y_{1:t}|\Psi_{1:t};\alpha ,\gamma ,\beta \right)\\ \; & =\arg\max_{\alpha ,\gamma ,\beta }\int_{R^{N}}p\left(y_{1:t}|\Psi_{1:t},f;\beta \right)p\left(f;\alpha ,\gamma \right)df. \end{array}$$

Then, the signal posterior given estimated hyperparameters is Gaussian:

(13) $$p\left(f|\Psi_{1:t},y_{1:t};\alpha_{t}^{\prime },\gamma_{t}^{\prime },\beta_{t}^{\prime }\right)=N\left(f\;|\;\mu_{t},C_{t}\right),$$

with mean vector and covariance matrix ([30], Section 10.6)

(14) $$\begin{array}{cc} \; & \mu_{t}=\beta_{t}^{\prime }C_{t}\Psi_{1:t}^{T}y_{1:t}, \end{array}$$

(15) $$\begin{array}{cc} \; & C_{t}={\left(\alpha_{t}^{\prime }I_{N}+\gamma_{t}^{\prime }L^{2n}+\beta_{t}^{\prime }\Psi_{1:t}^{T}\Psi_{1:t}\right)}^{-1}. \end{array}$$

The minimum mean square error (MMSE) or maximum a posteriori (MAP) estimation of the graph signal is $f_{t}^{\prime }=\mu_{t}$.

However, direct optimization of Equation (12) is intractable. We view f as a hidden variable, and introduce expectation maximization (EM) ([24], Section 9.4) in our algorithm to estimate α,γ,β, and thereby obtain the posterior distribution of f. To be concise, the subscripts *t* and 1:t are omitted in the introduction of our EM algorithm.

Consider the *l*-th EM iteration. In E step, hyperparameters α,γ,β are fixed at $\alpha_{l-1},\gamma_{l-1},$ $\beta_{l-1}$, and the signal posterior $p(f|\Psi ,y;\alpha_{l-1},\gamma_{l-1},\beta_{l-1})$ with mean $\mu_{l-1}$ and covariance $C_{l-1}$ is computed as in Equations (13)–(15).

In M step, α,γ,β are updated according to

(16) $$\begin{array}{cc} \alpha_{l},\gamma_{l},\beta_{l} & =\arg\max_{\alpha ,\gamma ,\beta }E_{f|\Psi ,y;\alpha_{l-1},\gamma_{l-1},\beta_{l-1}}\left[\ln p\left(y,f|\Psi ;\alpha ,\gamma ,\beta \right)\right]\\ \; & =\arg\max_{\alpha ,\gamma ,\beta }E_{f|\Psi ,y;\alpha_{l-1},\gamma_{l-1},\beta_{l-1}}\left[\ln p\left(y|\Psi ,f;\beta \right)+\ln p\left(f;\alpha ,\gamma \right)\right], \end{array}$$

where $E_{f|\Psi ,y;\alpha_{l-1},\gamma_{l-1},\beta_{l-1}}\left[\cdot \right]$ means the expectation is taken with respect to $p(f|\Psi ,y;\alpha_{l-1},$ $\gamma_{l-1},\beta_{l-1})$ computed at E step, the signal prior p(f;α,γ) is given in Equation (1), and the signal likelihood $p\left(y|\Psi ,f;\beta \right)=N\left(y\;|\;\Psi f,\beta^{-1}I\right)$ according to Equations (8) and (9).

For brevity, denote the expectation operator in Equation (16) as $E_{l-1}\left[\cdot \right]$, and the expectation value as $E_{l-1}$, which is also the objective value of the optimization problem. By direct calculations, we have

(17) $$\begin{array}{cc} E_{l-1}= & \frac{1}{2}\ln\left|\alpha I_{N}+\gamma L^{2n}\right|-\frac{\alpha }{2}E_{l-1}\left[f^{T}f\right]-\frac{\gamma }{2}E_{l-1}\left[f^{T}L^{2n}f\right]\\ \; & +\frac{M}{2}\ln\beta -\frac{\beta }{2}E_{l-1}\left[{\left(y-\Psi f\right)}^{T}\left(y-\Psi f\right)\right]+\mathrm{const}, \end{array}$$

where *M* is the sample size; “const” denotes terms that are independent of α,γ,β; and

(18) $$\begin{array}{cc} E_{l-1}\left[f^{T}f\right] & =E_{l-1}\left[{\left(f-\mu_{l-1}\right)}^{T}\left(f-\mu_{l-1}\right)+2\mu_{l-1}^{T}f-\mu_{l-1}^{T}\mu_{l-1}\right]\\ \; & =\ldots \left(C_{l-1}\right)+\mu_{l-1}^{T}\mu_{l-1}, \end{array}$$

(19) $$\begin{array}{cc} E_{l-1}\left[f^{T}L^{2n}f\right] & =E_{l-1}\left[{\left(f-\mu_{l-1}\right)}^{T}L^{2n}\left(f-\mu_{l-1}\right)+2\mu_{l-1}^{T}L^{2n}f-\mu_{l-1}^{T}L^{2n}\mu_{l-1}\right]\\ \; & =\ldots \left(L^{2n}C_{l-1}\right)+\mu_{l-1}^{T}L^{2n}\mu_{l-1}, \end{array}$$

(20) $$\begin{array}{cc} E_{l-1}\left[{\left(y-\Psi f\right)}^{T}\left(y-\Psi f\right)\right] & =E_{l-1}[{\left(y-\Psi \mu_{l-1}\right)}^{T}\left(y-\Psi \mu_{l-1}\right)\\ \; & -2{\left(y-\Psi \mu_{l-1}\right)}^{T}\Psi \left(f-\mu_{l-1}\right)\\ \; & +{\left(f-\mu_{l-1}\right)}^{T}\Psi^{T}\Psi \left(f-\mu_{l-1}\right)]\\ \; & ={\left(y-\Psi \mu_{l-1}\right)}^{T}\left(y-\Psi \mu_{l-1}\right)+\ldots \left(\Psi^{T}\Psi C_{l-1}\right). \end{array}$$

Note that $E_{l-1}$ is concave with respect to α,γ,β, see Appendix A. The maximization problem in Equation (16) can be solved by any standard tool for convex optimization. Here, the first order conditions of the optimization problem are analyzed to give some insights into the hyperparameter estimation, and then a possible efficient search method based on these conditions is provided in Appendix B.

Take the partial derivatives of $E_{l-1}$ with respect to α,γ,β, and set them to zero:

(21) $$\begin{array}{cc} \; & \frac{\partial E_{l-1}}{\partial \alpha }=\frac{1}{2}\ldots \left({\left(\alpha I_{N}+\gamma L^{2n}\right)}^{-1}\right)-\frac{1}{2}E_{l-1}\left[f^{T}f\right]=0, \end{array}$$

(22) $$\begin{array}{cc} \; & \frac{\partial E_{l-1}}{\partial \gamma }=\frac{1}{2}\ldots \left({\left(\alpha I_{N}+\gamma L^{2n}\right)}^{-1}L^{2n}\right)-\frac{1}{2}E_{l-1}\left[f^{T}L^{2n}f\right]=0, \end{array}$$

(23) $$\begin{array}{cc} \; & \frac{\partial E_{l-1}}{\partial \beta }=\frac{M}{2\beta }-\frac{1}{2}E_{l-1}\left[{\left(y-\Psi f\right)}^{T}\left(y-\Psi f\right)\right]=0. \end{array}$$

Note that $\mathrm{tr}({\left(\alpha I_{N}+\gamma L^{2n}\right)}^{-1})$, $\mathrm{tr}({\left(\alpha I_{N}+\gamma L^{2n}\right)}^{-1}L^{2n})$ and $\frac{M}{\beta }$ can be seen as the prior expectations of $f^{T}f$, $f^{T}L^{2n}f$ and $w^{T}w$ with respect to p(f;α,γ) and p(w;β). The M step actually looks for a group of hyperparameters that makes these prior expectations equal to their posterior ones. In this sense, the estimated hyperparameters agree with the observations.

Repeat the E and M steps as above until convergence, and we will obtain an MML estimation of unknown hyperparameters α,γ,β, together with a posterior of the graph signal f. This process is summarized in Algorithm 1. Although the estimated hyperparameters are not guaranteed to be globally optimal by MML, we find the performance good enough in experiments.
**Algorithm 1** Hyperparameter and signal estimation by EM.1:Initialize $\alpha_{0},\gamma_{0},\beta_{0}$.2:**for**l=1,2,⋯**do**3: Compute $\mu_{l-1},C_{l-1}$ as in Equations (14) and (15).4: Update $\alpha_{l},\gamma_{l},\beta_{l}$ according to Equation (16).5:**end for**

### 3.2. Sample Selection

Having been able to estimate the unknown hyperparameters from previous observations by EM, at each decision step, the subsequent sampling node can be selected using the latest estimated values of hyperparameters.

According to the uncertainty sampling (US) criterion in active learning ([25], Chapter 2), we should scan through all the nodes, and pick the one whose observed signal value we are most uncertain about as the next node to sample. Here, predictive variance is regarded as a measurement of uncertainty.

The predictive distribution of an observation *y* on vertex *s* with sampling vector $\psi^{T}=e_{s}^{T}$ given historical observations $\Psi_{1:t},y_{1:t}$ is

(24) $$p\left(y|\Psi_{1:t},y_{1:t},\psi^{T};\alpha_{t}^{\prime },\gamma_{t}^{\prime },\beta_{t}^{\prime }\right)=N\left(y\;|\;e_{s}^{T}\mu_{t},\;e_{s}^{T}C_{t}e_{s}+{\left(\beta_{t}^{\prime }\right)}^{-1}\right),$$

where $\mu_{t}$ and $C_{t}$ are the posterior mean and covariance of the graph signal f, respectively, given $\Psi_{1:t}$ and $y_{1:t}$. The predictive variance consists of two parts: estimative variance $e_{s}^{T}C_{t}e_{s}$ and noise variance ${\left(\beta_{t}^{\prime }\right)}^{-1}$, among which the latter is equal for all vertices. The next sampling node $s_{t+1}$ can be decided by

(25) $$s_{t+1}=\arg\max_{s\in V}e_{s}^{T}C_{t}e_{s}.$$

### 3.3. Sequential Sampling and Estimation

Finally, the complete procedure of the proposed sequential sampling and estimation algorithm for approximately bandlimited graph signals is given in Algorithm 2. At each step, first unknown hyperparameters are re-estimated by EM as in Section 3.1, and then the next node to sample is selected by US as in Section 3.2. The total number of sampling steps *T* is equal to the sampling budget.
**Algorithm 2** Sequential sampling and estimation of approximately bandlimited graph signals.1:Choose the first sampling node $s_{1}$ arbitrarily.2:**for**t=1,2,…**do**3: Sample the vertex $s_{t}$ and obtain an observation $y_{t}$.4: Update hyperparameters $\alpha_{t}^{\prime },\gamma_{t}^{\prime },\beta_{t}^{\prime }$ and signal posterior $\mu_{t},C_{t}$ by EM as in Algorithm 1.5: Select the next node to sample $s_{t+1}$ by US as in Equation (25).6:**end for**

Our sequential sampling strategy takes into account not only graph topology, but also previous observations, by both estimating hyperparameters based on historical data and selecting sampling nodes based on signal posterior. Making full use of historical observations and deciding which nodes to sample online, the proposed algorithm is able to cope with the situation where signal and noise distributions are not completely available in advance, and efficiently select samples to estimate the underlying graph signal.

The computational complexity of a decision step in the proposed algorithm is $O(n_{t}^{\mathrm{EM}}\bar{n_{t}^{\mathrm{bs}}}N^{3})$, where $n_{t}^{\mathrm{EM}}$ is the number of EM iterations, $\bar{n_{t}^{\mathrm{bs}}}$ is the average iterations of binary search in M step, and *N* is the graph size. The computational cost is acceptable in view of the performance improvement, especially in time-insensitive scenarios with large observation cost. The proposed method is able to efficiently select samples to give accurate estimation of signals thanks to the sequential framework with EM hyperparameter update, and the signal estimation is consistent.

## 4. Asymptotic Analysis

In the previous section, we have developed a sequential sampling and estimation algorithm for approximately bandlimited graph signals with an incomplete system model, where hyperparameter estimation and sample selection are performed in an alternating way. The performance of the proposed algorithm will be validated via simulation in the next section, where we will see its efficient sample selection and accurate signal estimation with limited sampling budget and little prior knowledge. In this section, we are to emphasize that the proposed algorithm improves limited-budget performance without sacrifice of consistency, which is not trivial for a sequential decision process.

Intuitively, as the number of observations grows, hyperparameters better fitting the true model will be picked out, really important nodes will be selected to sample, and finally an excellent estimation of the graph signal can be obtained. Here, we try to provide some theoretical support for this intuition. The asymptotic performance of the proposed algorithm is analyzed, and we state and prove the consistency of signal estimation in our method.

Before concentrating on the asymptotic performance of signal estimation, we first investigate the asymptotic behavior of sample selection in the proposed algorithm.

**Lemma** **1.** *Let $m_{i,t}$ denote the number of times vertex i is sampled up to step t. If $\lim_{t\to \infty }\frac{1}{t}\frac{tr(\alpha_{t}^{\prime }I_{N}+\gamma_{t}^{\prime }L^{2n})}{N\beta_{t}^{\prime }}$=0,*

$$\exists \;\delta >0,\;\underset{t\to \infty }{lim inf}\min_{i\in V}\frac{m_{i,t}}{t}>\delta .$$

That is, as t→∞, all the nodes of the graph will be sampled again and again, each maintaining a sampling ratio greater than δ, including the lowest one. A proof of Lemma 1 is given in Appendix C. Then, two direct corollaries follow.

**Corollary** **1.** *∀i∈V, ∃δ>0, $\underset{t\to \infty }{lim inf}\frac{m_{i,t}}{t}>\delta$.*

**Corollary** **2.** *∀i∈V, $\lim_{t\to \infty }m_{i,t}=\infty$.*

Based on them, we are now to state our main theorem on the consistency of signal estimation in the proposed algorithm.

**Theorem** **2.** *The signal posterior $p(f|\Psi_{1:t},y_{1:t};\alpha_{t}^{\prime },\gamma_{t}^{\prime },\beta_{t}^{\prime })$ is consistent at the true value $f^{*}$, if EM converges at MML hyperparameters satisfying the condition in Lemma 1.*

The consistency of signal posterior here means, as t→∞, the posterior distribution will become a point mass at the true value of the graph signal. A proof of Theorem 2 is provided in Appendix D. Then, we have the following corollary.

**Corollary** **3.** *The MMSE/MAP estimation $f_{t}^{\prime }=\mu_{t}$ is consistent at $f^{*}$ under the conditions in Theorem 2.*

It ensures that under specific conditions, as the sample size grows infinitely large, the MMSE/MAP estimation can get arbitrarily close to the true signal value, i.e., the graph signal can be estimated with arbitrary accuracy. This guarantees the asymptotic performance of the proposed algorithm in signal estimation.

The condition $\lim_{t\to \infty }\frac{1}{t}\frac{\mathrm{tr}(\alpha_{t}^{\prime }I_{N}+\gamma_{t}^{\prime }L^{2n})}{N\beta_{t}^{\prime }}=0$ is satisfied when $\frac{\alpha_{t}^{\prime }}{\beta_{t}^{\prime }}=o\left(t\right)$, $\frac{\gamma_{t}^{\prime }}{\beta_{t}^{\prime }}=o\left(t\right)$, or more sufficiently, if $\underset{t\to \infty }{lim sup}\alpha_{t}^{\prime }<\infty$, $\underset{t\to \infty }{lim sup}\gamma_{t}^{\prime }<\infty$, $\underset{t\to \infty }{lim inf}\beta_{t}^{\prime }>0$, which ensures that the estimated signal energy and bandwidth do not decrease to zero, and the estimated noise variance keeps finite. We did not observe any violation of these conditions in plenty of experiments, where the true graph signals do not have all same values, and all observations are finite.

## 5. Experiments

In this section, the finite-time performance of the proposed algorithm is validated via a series of simulation experiments, on both synthetic and real-world data.

The performance of the proposed algorithm is compared to the following methods:

- RndEM: Select sampling nodes randomly and uniformly, and estimate hyperparameters and the signal using the proposed EM algorithm.
- OrcUS: Suppose that the hyperparameters are known in advance (the “oracle”). Select sampling nodes by US, and estimate the signal by MMSE/MAP.
- Anis2016: A heuristic sampling algorithm proposed in Anis et al. [7] to maximize the cut-off frequency such that the sampling set is a uniqueness set for the bandlimited subspace. The graph signal is recovered in the bandlimited subspace by least squares. The estimation order *k* of cut-off frequency is set to k=4.
- Perraudin2018: A nonuniform random sampling method proposed in Perraudin et al. [21] with probability relevant to local uncertainty. Inpainting is done by minimizing total variation. The kernel in local uncertainty is $\hat{g}\left(\lambda \right)=\exp\left(-\tau \lambda \right)$ where τ=8.
- Sakiyama2019: In this method by Sakiyama et al. [31], the graph signal is recovered by a linear combination of localization operators at the sampled nodes. The sampling set is designed such that the energy of operator at each node is large and meanwhile the overlapping areas are small. The kernel in localization operator is $\hat{g}\left(\lambda \right)=\exp\left(-\tau \lambda \right)$ where τ=0.5. The Chebyshev polynomial approximation order is P=12, and the signal recovery order is k=12.

RndEM and OrcUS are references to verify the effectiveness of the proposed algorithm. Anis2016, Perraudin2018, Sakiyama2019 are three state-of-the-art methods that do not directly rely on signal model. For Anis2016, the benefit of further increasing *k* is not noticeable in our experiments, but the computations will become less numerically stable. For Perraudin2018 and Sakiyama2019, we use the same kernel type as in the original papers, and the parameters are chosen by experiments to adjust to our settings.

The normalized $l_{2}$-norm estimation error

(26) $$e=\frac{∥f^{\prime }-f^{*}{∥}_{2}}{∥f^{*}{∥}_{2}}$$

is taken as a performance index, where $f^{\prime }$ is the estimated signal, and $f^{*}$ is the true value.

### 5.1. Simulations on Synthetic Data

Three graphs of representative types that have widespread applications in the real world are used for testing in our experiments:

- $G_{1}$: A random geometric graph with N=100 vertices randomly placed in a 1-by-1 square, and edge weights assigned via a thresholded Gaussian kernel
  (27) $$w_{ij}=\left\{\begin{array}{cc} \exp\;\left(-\frac{d_{ij}^{2}}{2\sigma^{2}}\right), & \mathrm{if}\;d_{ij}<r\\ 0, & \mathrm{otherwise} \end{array}\right.$$
  where $d_{ij}$ is the Euclid distance between vertex *i* and vertex *j*, r=0.2 and σ=0.1.
- $G_{2}$: A small world graph with N=100 nodes, generated by the Watts–Strogatz model [32] with mean node degree 6 and rewiring probability 0.2.
- $G_{3}$: A scale-free graph with N=100 nodes, generated by the Barabási–Albert model [33,34] with 1 initial node, each newly added node connected to 1 existing node, and connecting probabilities proportional to the degrees of existing nodes.

**Figure 1:**
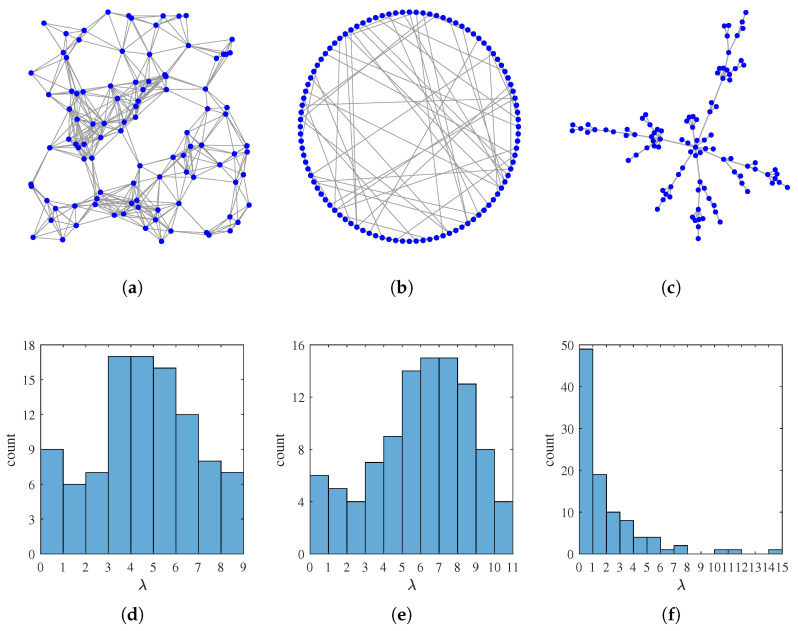
The three graphs used in our simulations, and the histograms of their eigenvalue distributions. (**a**) $G_{1}$, a random geometric graph and (**d**) its eigenvalue distribution. (**b**) $G_{2}$, a Watts–Strogatz small world graph and (**e**) its eigenvalue distribution. (**c**) $G_{3}$, a Barabási–Albert scale-free graph and (**f**) its eigenvalue distribution.

The graph topologies as well as their eigenvalue distributions are visualized in Figure 1. Due to limited space, we will mainly display and analyze our experimental results on $G_{1}$ in detail, whereas example results on $G_{2}$ and $G_{3}$ are shown at the end to demonstrate the topology adaptability.

Graph signals are generated from the ABL prior distribution Equation (1). Moreover, observation noise follows i.i.d. zero-mean Gaussian distribution Equation (6). A variety of hyperparameters are considered, in order to cover ABL graph signals with different bandwidth, strictness of bandlimitedness and energy level, and scenarios with different signal-noise ratio (SNR). SNR here considering random ABL graph signal is defined as

(28) $${SNR}_{r}=10\mathrm{lg}\frac{\sum_{k=1}^{N}\;1/\left(\alpha +\gamma \lambda_{k}^{2n}\right)}{N/\beta }.$$

For each setting, 100 graph signals are generated to evaluate the average performance of each method. The average estimation errors under different sample sizes are computed and plotted.

**Figure 2:**
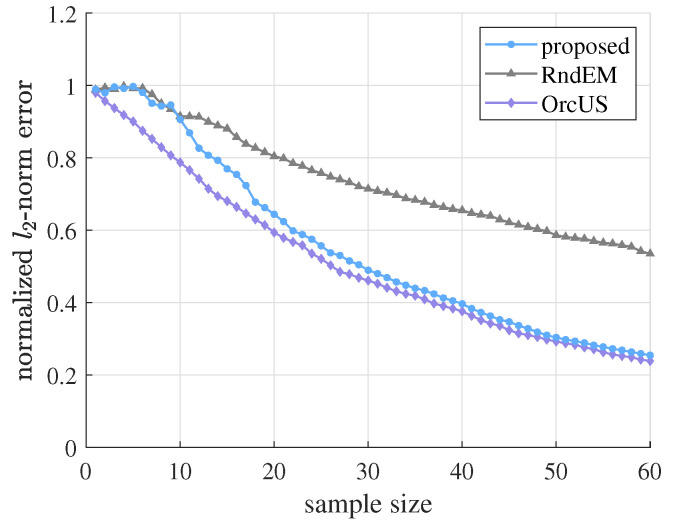
Estimation error with growing sample size of the proposed algorithm versus RndEM and OrcUS on $G_{1}$. Hyperparameters are n=2, $\lambda_{c}=2$, g=1, and ${SNR}_{r}=15\;$dB.

First of all, we compare the performance of the proposed algorithm with RndEM and OrcUS on $G_{1}$. Hyperparameters are set to n=2, $\lambda_{c}=2$, g=1, and ${SNR}_{r}=15\;$dB. As shown in Figure 2, although in the initial stage, the estimation error of the proposed algorithm is similar to RndEM, as the sample size grows, our performance gradually approaches that of OrcUS. This result is as expected, as hyperparameter estimation gets better with growing sample size, which can lead to more efficient sample selection and more accurate signal estimation. Our superiority to RndEM (EM estimation without US sampling) demonstrates the effectiveness of choosing sampling nodes by US. The performance gap between our method and OrcUS (US sampling with known hyperparameters) tends to disappear when samples are abundant, also supporting the validity of hyperparameter estimation by EM.

**Figure 3:**
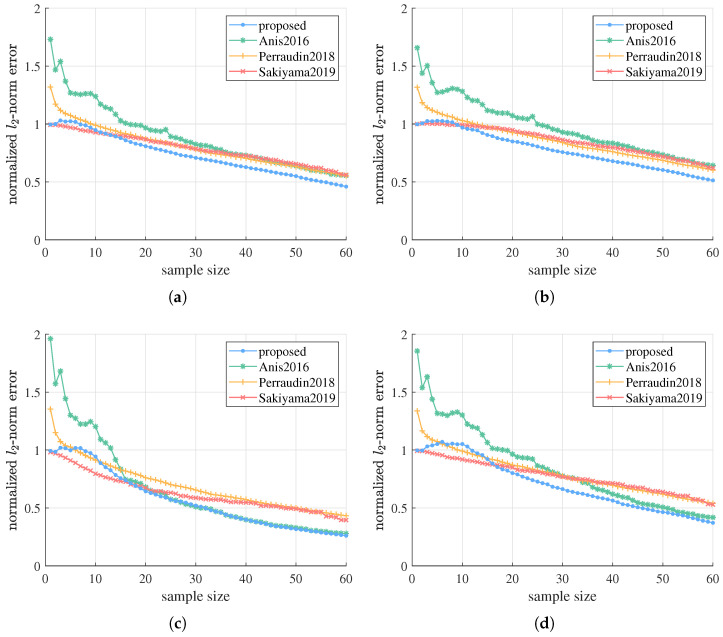
Estimation errors of the proposed algorithm versus Anis2016, Perraudin2018, and Sakiyama2019 on $G_{1}$ with different signal bandwidth and strictness of bandlimitedness, i.e., different *n* and $\lambda_{c}$. We fix g=1 and ${SNR}_{r}=15$ dB. (**a**) $n=1,\;\lambda_{c}=2$. (**b**) $n=1,\;\lambda_{c}=3$. (**c**) $n=2,\;\lambda_{c}=2$. (**d**) $n=2,\;\lambda_{c}=3$.

Subsequently, the proposed algorithm is compared to three existing sampling and estimation methods—Anis2016, Perraudin2018, and Sakiyama2019—in scenarios without much prior knowledge about signal and noise model. A series of experiments with different hyperparameters are done on $G_{1}$ to test and compare their performance in all kinds of settings. In the first experiment, we fix g=1, ${SNR}_{r}=15\;$dB, and repeat the simulation with n=1,2, $\lambda_{c}=2,3$, corresponding to ABL graph signals with different bandwidth and strictness of bandlimitedness. Simulation results are depicted in Figure 3. From the graphics we can see, the proposed algorithm can obtain better or competitive estimation performance compared to state-of-the-art methods on ABL graph signals with various properties. For Perraudin2018 and Sakiyama2019, the proposed method significantly outperforms them after a small number of initial samples in all the four settings. As for Anis2016, when *n* is small or $\lambda_{c}$ is large, which means the signal is far from strictly bandlimited or occupies large bandwidth, we need far fewer samples than Anis2016 to recover the signal with tolerable error. This is because our method estimates the graph signal at all frequencies from the beginning thanks to the Bayesian framework, and our signal prior with hyperparameters estimated from data can better fit ABL graph signals with different properties. Therefore, the proposed algorithm is more robust against different signal properties and is able to obtain better estimation results in most cases.

**Figure 4:**
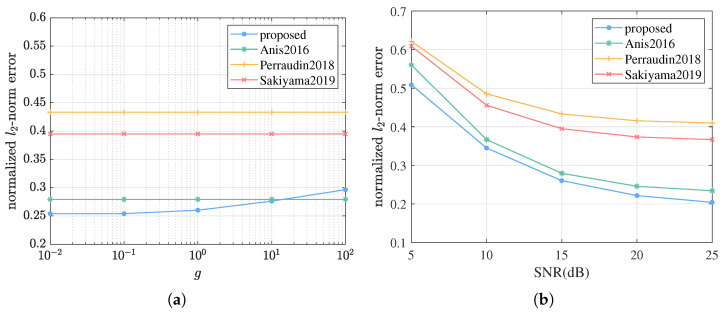
Estimation errors of the proposed algorithm versus Anis2016, Perraudin2018, and Sakiyama2019 on $G_{1}$ with different signal energy level and signal–noise ratio, i.e., different *g* and ${SNR}_{r}$. We fix n=2, $\lambda_{c}=2$. The sampling size is set to M=60. (**a**) *g* is changed from 0.01 to 100, while ${SNR}_{r}$ is fixed at 15 dB. (**b**) ${SNR}_{r}$ is changed from 5 dB to 25 dB, while *g* is fixed at 1.

We continue to investigate our performance for ABL graph signals with different energy level as well as in scenarios with different signal–noise ratio. We fix n=2, $\lambda_{c}=2$. When changing *g* from 0.01 to 100, ${SNR}_{r}$ is fixed at 15 dB. When changing ${SNR}_{r}$ from 5 dB to 25 dB, *g* is fixed at 1. Sampling size is set to M=60. According to Figure 4a, the proposed algorithm gets better performance for graph signals with lower energy. For one thing, even though the estimated variance in the signal prior also becomes larger when the signal energy is larger, the zero-mean Gaussian prior still prefers signals with lower energy. For another, as the variance gets larger, the signal prior becomes flatter, and thus weaker, in the sense that it has less preference among different signals. Therefore, more observations are needed to reach the same estimation accuracy. Fortunately, this problem can be overcome by adaptive rescaling, dividing the observed signal values by a sufficiently large number, as long as we know the rough order of magnitudes of the signal energy. As for SNR, from Figure 4b, although lower SNR may result in worse performance of the proposed algorithm, our method keeps outperforming the others when samples are adequate.

**Figure 5:**
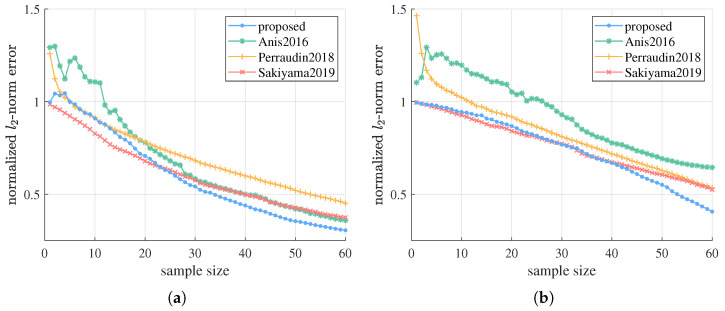
Estimation errors of the proposed algorithm versus Anis2016, Perraudin2018, and Sakiyama2019 on $G_{2}$ and $G_{3}$. (**a**) Results on $G_{2}$ with n=2, $\lambda_{c}=3$, g=1, ${SNR}_{r}=15$ dB. (**b**) Results on $G_{3}$ with n=2, $\lambda_{c}=1$, g=1, ${SNR}_{r}=15$ dB.

Then, example simulation results on $G_{2}$ and $G_{3}$ are given in Figure 5. n=2, g=1, ${SNR}_{r}=15$ dB. $\lambda_{c}=3$ for $G_{2}$, and $\lambda_{c}=1$ for $G_{3}$. The proposed algorithm also reaches higher estimation accuracy than the other methods with enough sampling budget. This demonstrates the robustness of the proposed algorithm against different graph topologies.

From the simulation results above, without much prior knowledge, the proposed algorithm can adjust to ABL graph signals with different bandwidth, strictness of bandlimitedness, energy level, and SNR. In most cases, exceeding a small number of initial samples, the proposed algorithm is able to estimate the graph signals with higher accuracy than the existing methods.

Besides the validation of the limited-budget performance, we also verify the consistency of the proposed algorithm by a simulation experiment. We generate an ABL graph signal on $G_{1}$ with n=2, $\lambda_{c}=2$, and g=1, and the SNR is set to 15dB. To observe the asymptotic performance, the maximum sample size is set to $10^{5}$, which is $10^{3}$ times the graph size. We repeat the proposed algorithm on the graph signal for 50 times to see the mean as well as the variance of the estimation errors.

**Figure 6:**
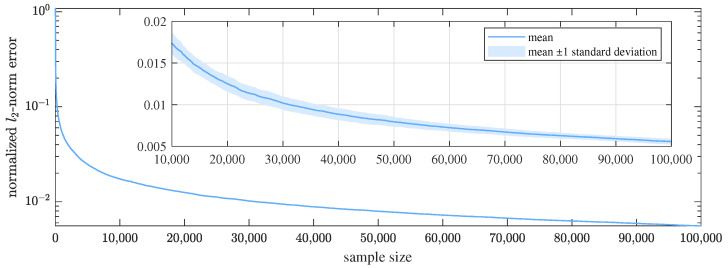
Result of the consistency verification experiment. The graph signal is on $G_{1}$ generated with n=2, $\lambda_{c}=2$, g=1, and the observation SNR is 15dB. The curve denotes the mean of the normalized $l_{2}$-norm error, and the region corresponds to ± 1 standard deviation around the mean. In the overview figure, the y-axis is in logarithmic scale to better display the decrease of the mean estimation error, whereas in the partial enlargement, it is linear to present the decline of the standard deviation.

As shown in Figure 6, the mean normalized $l_{2}$-norm error tends to decrease continuously as the sample size grows, and has decreased to about $5\;\;\times \;\;10^{-3}$ when the sample size reaches $10^{5}$. The standard deviation also tends to decline with growing sample size, which is around $3\;\times \;10^{-4}$ with $10^{5}$ samples. Other ABL graph signals also have similar results. This agrees with the consistency of the proposed algorithm, which has been stated and theoretically proved in Section 4.

### 5.2. A Real-World Example of Temperature Sensor Network

As an application example, we apply the proposed algorithm to the air temperatures measured by weather stations in China [35]. This example shows a potential application of the proposed algorithm in sensor selection.

We construct a *K*-nearest neighbor (*K*-NN) graph from the N=381 weather stations with K=5, where each node corresponds to a weather station, and the great-circle distances between stations are considered in *K*-NN search. Edge weights are assigned via a Gaussian kernel:

(29) $$w_{ij}=\exp\;\left(-\frac{d_{ij}^{2}}{2\sigma^{2}}\right)\;\mathrm{for}\;\left(i,j\right)\in E,$$

where $d_{ij}$ is the great-circle distance between station *i* and station *j* in degree, and σ=2.

**Figure 7:**
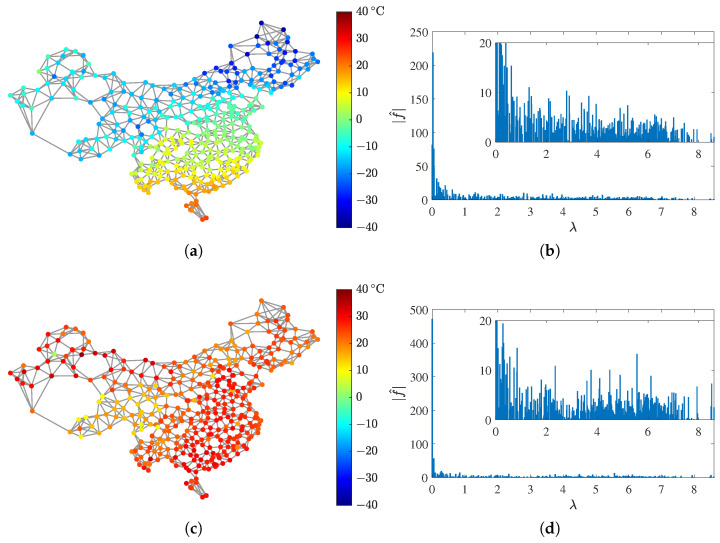
Two graph signals of air temperatures measured by 381 weather stations in China, and their spectra. (**a**) $f_{1}$ at 0:00 on 1 January 2020, and (**b**) its spectrum. (**c**) $f_{2}$ at 12:00 on 1 July 2020, and (**d**) its spectrum.

Then, the air temperatures measured by these weather stations can be seen as a graph signal. We consider two such signals in our experiment: $f_{1}$ at 0:00 on 1 January 2020, which is a winter night, and $f_{2}$ at 12:00 on 1 July 2020, which is a summer noon. The two graph signals and their spectra are visualized in Figure 7. As expected, the signals are smooth in the vertex domain and approximately bandlimited in the frequency domain.

We apply the proposed sequential sampling and estimation algorithm to these graph signals. Additive i.i.d. zero-mean Gaussian observation noise following Equation (6) is added, where ${SNR}_{d}=10\mathrm{lg}\frac{f^{T}f}{N/\beta }$ is 15 dB. The known hyperparameter *n* in the signal prior Equation (1) is set to 1/2, which means a smoothness prior Equation (3) is considered. The observed signal values are divided by 100 as preprocessing, and accordingly the estimated graph signal is multiplied by 100 at output. The performance of the proposed algorithm is compared to Anis2016, Perraudin2018 and Sakiyama2019. As our first sampling node is chosen at random, Perraudin2018 is a random sampling method, and the observation noises also introduce randomness, we test each method on the same signal for 20 times, and compare their average estimation errors.

**Figure 8:**
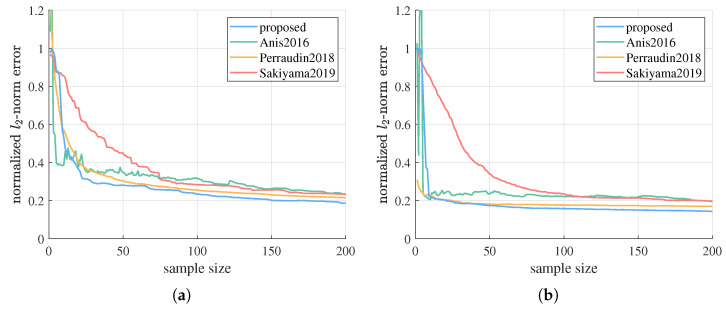
Estimation errors of the two temperature graph signals by the proposed algorithm versus Anis2016, Perraudin2018, and Sakiyama2019. (**a)** Results on $f_{1}$. (**b**) Results on $f_{2}$.

As shown in Figure 8, although the proposed algorithm needs some initial samples to “warm up”, after that the estimation error decreases rapidly and surpass Anis2016, Perraudin2018, and Sakiyama2019 in very short time, and we keep leading as the sample size further grows. This is because our sequential method with unknown hyperparameters requires a certain number of samples to have a relatively proper estimation of the signal and noise distributions. Then, based on the latest signal bandwidth, energy level, and noise precision estimated from data, we are able to choose the subsequent sampling nodes more efficiently and estimate the graph signal more accurately than other methods that take no account of such signal and noise properties.

## 6. Conclusions

In this paper, a sequential sampling and estimation algorithm is proposed for approximately bandlimited graph signals without complete prior knowledge about signal and noise properties. In the proposed algorithm, hyperparameter estimation by EM based on historical observations and sample selection by US with latest estimated hyperparameters are done in an alternating way. We prove in theory the consistency of signal estimation in the proposed algorithm under specific conditions. Finite-time performance of the proposed algorithm is validated by a series of simulation results. The proposed method provides a novel and practical sequential framework for graph signal sampling with unknown hyperparameters in the system model, and the experiment on temperature data shows our potential application in sensor selection on sensor networks with smooth measurements over the topologies. Other potential applications include opinion prediction in social networks assuming that people with strong social connection are likely to hold similar opinions, and active semisupervised learning tasks on similarity graphs.

## Figures and Tables

**Figure A1:**
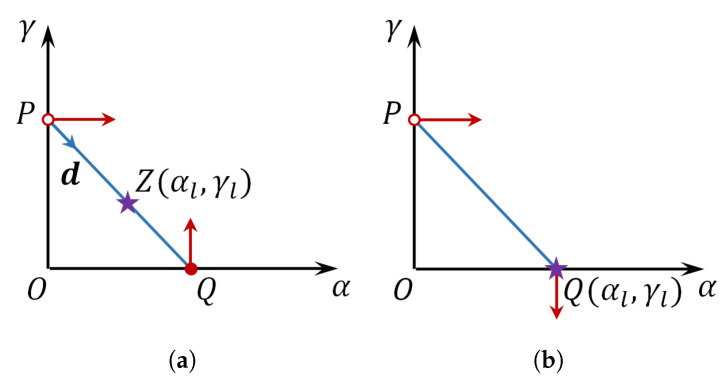
Two cases of M-step update of α,γ. (**a**) Case I when Δ>0: The solution point $\left(\alpha_{l},\gamma_{l}\right)$ locates on PQ between P and Q. (**b**) Case II when Δ≤0: The endpoint Q is the solution for $\left(\alpha_{l},\gamma_{l}\right)$.

## Data Availability

No new data were created or analyzed in this study. Data sharing is not applicable to this article.

## Appendix A. Concavity of Objective Function in M Step

The objective function in Equation (16) is concave with respect to α,γ,β, as the Hessian matrix

(A1) $$\begin{array}{cc} H=\left[\begin{array}{ccc} \frac{\partial^{2}E_{l-1}}{\partial \alpha^{2}}\;\; & \;\;\frac{\partial^{2}E_{l-1}}{\partial \alpha \partial \gamma }\;\; & \;\;\frac{\partial^{2}E_{l-1}}{\partial \alpha \partial \beta }\\ \frac{\partial^{2}E_{l-1}}{\partial \gamma \partial \alpha }\;\; & \;\;\frac{\partial^{2}E_{l-1}}{\partial \gamma^{2}}\;\; & \;\;\frac{\partial^{2}E_{l-1}}{\partial \gamma \partial \beta }\\ \frac{\partial^{2}E_{l-1}}{\partial \beta \partial \alpha }\;\; & \;\;\frac{\partial^{2}E_{l-1}}{\partial \beta \partial \gamma }\;\; & \;\;\frac{\partial^{2}E_{l-1}}{\partial \beta^{2}} \end{array}\right]\; & =\left[\begin{array}{ccc} -\frac{1}{2}\sum_{k=1}^{N}\frac{1}{{\left(\alpha +\gamma \lambda_{k}^{2n}\right)}^{2}}\;\; & \;\;-\frac{1}{2}\sum_{k=1}^{N}\frac{\lambda_{k}^{2n}}{{\left(\alpha +\gamma \lambda_{k}^{2n}\right)}^{2}}\;\; & \;\;0\\ -\frac{1}{2}\sum_{k=1}^{N}\frac{\lambda_{k}^{2n}}{{\left(\alpha +\gamma \lambda_{k}^{2n}\right)}^{2}}\;\; & \;\;-\frac{1}{2}\sum_{k=1}^{N}\frac{\lambda_{k}^{4n}}{{\left(\alpha +\gamma \lambda_{k}^{2n}\right)}^{2}}\;\; & \;\;0\\ 0\;\; & \;\;0\;\; & \;\;-\frac{N}{2\beta^{2}} \end{array}\right]\\ \; & =-\frac{1}{2}\sum_{k=1}^{N}\frac{1}{{\left(\alpha +\gamma \lambda_{k}^{2n}\right)}^{2}}\left[\begin{array}{c} 1\\ \lambda_{k}^{2n}\\ 0 \end{array}\right]\left[\begin{array}{c} 1\;\;\lambda_{k}^{2n}\;\;0 \end{array}\right]+\left[\begin{array}{ccc} 0\;\; & \;\;0\;\; & \;\;0\\ 0\;\; & \;\;0\;\; & \;\;0\\ 0\;\; & \;\;0\;\; & \;\;-\frac{N}{2\beta^{2}} \end{array}\right] \end{array}$$

is negative semi-definite for all α,γ,β.

## Appendix B. Search Method for Optimization Problem in M Step

To maximize $E_{l-1}$ to solve Equation (16), take the partial derivatives with respect to α,γ,β and set them to zero as in Equations (21)–(23).

From Equation (23), the M-step update of β has closed form

(A2) $$\beta_{l}=\frac{M}{E_{l-1}\left[{\left(y-\Psi f\right)}^{T}\left(y-\Psi f\right)\right]},$$

which is independent of the update of α,γ.

As for α,γ, as the approximately bandlimited prior Equation (1) requires α,γ>0, if Equations (21) and (22) hold for some α,γ>0, this (α,γ) is the solution for $\left(\alpha_{l},\gamma_{l}\right)$; otherwise, $\left(\alpha_{l},\gamma_{l}\right)$ should be determined using gradient information based on concavity.

Combining Equations (21) and (22) to have $\alpha \frac{\partial E_{l-1}}{\partial \alpha }+\gamma \frac{\partial E_{l-1}}{\partial \gamma }=0$, we get

(A3) $$\alpha E_{l-1}\left[f^{T}f\right]+\gamma E_{l-1}\left[f^{T}L^{2n}f\right]=N,$$

which constrains (α,γ) on a straight line in the α-γ plane. Only the segment PQ in the first quadrant is considered as shown in Figure A1 to ensure α,γ>0. The endpoint *P* has coordinates $\left(0,\frac{N}{E_{l-1}\left[f^{T}L^{2n}f\right]}\right)$, and the endpoint *Q* is at $\left(\frac{N}{E_{l-1}\left[f^{T}f\right]},0\right)$.

Define the gradient of $E_{l-1}$ with respect to α,γ as $g={\left[\frac{\partial E_{l-1}}{\partial \alpha },\frac{\partial E_{l-1}}{\partial \gamma }\right]}^{T}\;$. Any point *A* on PQ satisfies ${g|}_{A}⊥OA$. The gradient approaching *P*

(A4) $${g|}_{(\alpha ,\gamma )\to P}={\left[+\infty ,0\right]}^{T}$$

is always rightward as illustrated in Figure A1. Moreover, the gradient at *Q*

(A5) $${g|}_{Q}={\left[0,\frac{1}{2}\Delta \right]}^{T}\;\;,\;\mathrm{where}\;\Delta =\frac{\ldots (L^{2n})}{N}E_{l-1}\left[f^{T}f\right]-E_{l-1}\left[f^{T}L^{2n}f\right],$$

is either upward or downward, depending on the sign of Δ.

If Δ>0, the gradient at *Q* is upward as in Figure A1a. Let d be the unit vector of direction PQ. Consider the directional derivative

(A6) $$\begin{array}{c} \frac{\partial E_{l-1}}{\partial d}=\langle g,d\rangle \propto \frac{\partial E_{l-1}}{\partial \alpha }E_{l-1}\left[f^{T}L^{2n}f\right]-\frac{\partial E_{l-1}}{\partial \gamma }E_{l-1}\left[f^{T}f\right]. \end{array}$$

$\frac{\partial E_{l-1}}{\partial d}{|}_{(\alpha ,\gamma )\to P}>0$ and $\frac{\partial E_{l-1}}{\partial d}{|}_{Q}<0$ are different in sign. By continuity of $\frac{\partial E_{l-1}}{\partial d}$, there exists a zero-crossing *Z* of $\frac{\partial E_{l-1}}{\partial d}$ on PQ between *P* and *Q*. Moreover, by concavity of $E_{l-1}$, $\frac{\partial E_{l-1}}{\partial d}$ is monotonous along PQ. The zero-crossing *Z* can be found using, for example, binary search. Notice that ${g|}_{Z}$ satisfies both ${g|}_{Z}⊥PQ$ and ${g|}_{Z}⊥OZ$, so ${g|}_{Z}=0$, i.e., Equations (21) and (22) hold at *Z*. Therefore, *Z* is the solution point for $\left(\alpha_{l},\gamma_{l}\right)$.

Otherwise, if Δ≤0, the gradient at *Q* is zero or downward as in Figure A1b. By concavity of $E_{l-1}$, the endpoint *Q* is the optimal point in the first quadrant. $\gamma_{l}$ is set to 0, and α is updated by

(A7) $$\alpha_{l}=\frac{N}{E_{l-1}\left[f^{T}f\right]}.$$

In this case, $p\left(f;\alpha_{l},\gamma_{l}\right)=N\left(f\;|\;0,\alpha_{l}^{-1}I_{N}\right)$, signal values on different nodes become i.i.d., and only the total energy of the graph signal is restricted.

## Appendix C. Proof of Lemma 1

**Proof.** Let ${\left\{m_{(r),t}\right\}}_{r=1}^{N}$ be the order statistics of ${\left\{m_{i,t}\right\}}_{i\in V}$ satisfying $m_{(1),t}\leq \cdots \leq m_{(N),t}$, and denote the sorted vertex indices by ${\left\{i_{(r),t}\right\}}_{r=1}^{N}$. Consider $\eta_{i,t}=\frac{m_{i,t}}{t}$, and $\eta_{(r),t}=\frac{m_{(r),t}}{t}$. Define

(A8) $$R=\min\left\{r\;|\;\exists \;\delta >0,\;\underset{t\to \infty }{lim inf}\eta_{(r),t}>\delta \right\}-1.$$

Note that $\underset{t\to \infty }{lim inf}\eta_{(r),t}$ always exists as $\eta_{i,t}$ is bounded in [0,1]. Moreover, the set is not empty as $\eta_{(N),t}=\max_{i\in V}\eta_{i,t}\geq \frac{1}{N}$. So 0≤R≤N−1. Suppose that R≥1. Let $\rho_{t}=\eta_{(R),t}$. We are to show that $\underset{t\to \infty }{lim inf}\rho_{t}\geq \underline{\rho }$ for some $\underline{\rho }>0$, which contradicts the definition of *R*. Then we will arrive at an assertion R=0, and lemma 1 is proved. Define $P_{t}=\frac{1}{t}C_{t}=\frac{1}{t}A_{t}^{\prime }+\beta_{t}^{\prime }\sum_{i\in V}\eta_{i,t}e_{i}e_{i}^{T}$, where $A_{t}^{\prime }=\alpha_{t}^{\prime }I_{N}+\gamma_{t}^{\prime }L^{2n}$. The sampling strategy Equation (25) can also be rewritten as

(A9) $$s_{t+1}=\arg\max_{i\in V}e_{i}^{T}P_{t}^{-1}e_{i}.$$

Define $I_{t}=\left\{i_{(r),t}\;|\;r\geq R+1\right\}$, and $I_{t}^{c}=\left\{i_{(r),t}\;|\;r\leq R\right\}$. By definition, there exists δ>0 and T<∞, for all t>T and $i\in I_{t}$, it holds $\eta_{i,t}>\delta$. As $A_{t}^{\prime }$ is positive definite,

(A10) $$\forall \;t>T,\;\forall \;i\in I_{t},\;e_{i}^{T}P_{t}^{-1}e_{i}\leq e_{i}^{T}{\left(\beta_{t}^{\prime }\sum_{j\in V}\eta_{j,t}e_{j}e_{j}^{T}\right)}^{\;\;†}\;e_{i}=\frac{1}{\beta_{t}^{\prime }\eta_{i,t}}<\frac{1}{\beta_{t}^{\prime }\delta },$$

where ${\left(\cdot \right)}^{†}$ denotes the pseudo-inverse of a matrix. Consider $i\in I_{t}^{c}$. Define

(A11) $$\begin{array}{cc} \; & Q_{t}^{\left(1\right)}=\beta_{t}^{\prime }\left(1-\rho_{t}\right)\sum_{i\in I_{t}}e_{i}e_{i}^{T}, \end{array}$$

(A12) $$\begin{array}{cc} \; & Q_{t}^{\left(2\right)}=\frac{1}{t}A_{t}^{\prime }+\beta_{t}^{\prime }\rho_{t}\sum_{i\in V}e_{i}e_{i}^{T}. \end{array}$$

As $P_{t}⪯Q_{t}^{\left(1\right)}+Q_{t}^{\left(2\right)}=\frac{1}{t}A_{t}^{\prime }+\beta_{t}^{\prime }\left(\sum_{i\in I_{t}}\;e_{i}e_{i}^{T}+\rho_{t}\;\sum_{i\in I_{t}^{c}}\;e_{i}e_{i}^{T}\right)$,

(A13) $$\begin{array}{cc} \max_{i\in I_{t}^{c}}e_{i}^{T}P_{t}^{-1}e_{i} & \geq \max_{i\in I_{t}^{c}}e_{i}^{T}{\left(Q_{t}^{\left(1\right)}+Q_{t}^{\left(2\right)}\right)}^{-1}e_{i}\\ \; & =\max_{i\in I_{t}^{c},z\in R^{N}}\;2e_{i}^{T}z-z^{T}\left(Q_{t}^{\left(1\right)}+Q_{t}^{\left(2\right)}\right)z\\ \; & \geq \max_{i\in I_{t}^{c},z\in N\left(Q_{t}^{\left(1\right)}\right)}\;2e_{i}^{T}z-z^{T}Q_{t}^{\left(2\right)}z, \end{array}$$

where $N(Q_{t}^{\left(1\right)})$ is the null space of $Q_{t}^{\left(1\right)}$. Denote the maximum value in the last line, also a lower bound of $\max_{i\in I_{t}^{c}}e_{i}^{T}P_{t}^{-1}e_{i}$, as $L_{t}$, and the maximizers as $i_{t}^{*}$, $z_{t}^{*}$. On one hand, when *i* is set to $i_{t}^{*}$, we have

(A14) $$L_{t}=\max_{z\in N(Q_{t}^{\left(1\right)})}\;2e_{i_{t}^{*}}^{T}z-z^{T}Q_{t}^{\left(2\right)}z.$$

By Lagrange multiplier method,

(A15) $$\begin{array}{cc} \; & z_{t}^{*}={\left(Q_{t}^{\left(2\right)}\right)}^{-1}\left(I_{N}-Q_{t}^{\left(1\right)}{\left(Q_{t}^{\left(1\right)}{\left(Q_{t}^{\left(2\right)}\right)}^{-1}Q_{t}^{\left(1\right)}\right)}^{†}Q_{t}^{\left(1\right)}{\left(Q_{t}^{\left(2\right)}\right)}^{-1}\right)e_{i_{t}^{*}}, \end{array}$$

(A16) $$\begin{array}{cc} \; & L_{t}=2e_{i_{t}^{*}}^{T}z_{t}^{*}-{\left(z_{t}^{*}\right)}^{T}Q_{t}^{\left(2\right)}z_{t}^{*}=e_{i_{t}^{*}}^{T}z_{t}^{*}={\left(z_{t}^{*}\right)}^{T}Q_{t}^{\left(2\right)}z_{t}^{*}. \end{array}$$

On the other hand, when z is fixed at $z_{t}^{*}$, we know

(A17) $$e_{i_{t}^{*}}^{T}z_{t}^{*}=\max_{i\in I_{t}^{c}}e_{i}^{T}z_{t}^{*}.$$

We further assert that

(A18) $$e_{i_{t}^{*}}^{T}z_{t}^{*}=\max_{i\in I_{t}^{c}}\left|e_{i}^{T}z_{t}^{*}\right|.$$

Otherwise, there exists $j\in I_{t}^{c}$ such that $-e_{j}^{T}z_{t}^{*}>e_{i_{t}^{*}}^{T}z_{t}^{*}$. This will lead to a contradiction $2e_{j}^{T}\left(-z_{t}^{*}\right)-{\left(-z_{t}^{*}\right)}^{T}Q_{t}^{\left(2\right)}\left(-z_{t}^{*}\right)>L_{t}$. Therefore,

(A19) $$\begin{array}{cc} {\left(e_{i_{t}^{*}}^{T}z_{t}^{*}\right)}^{2} & =\max_{i\in I_{t}^{c}}\;{\left(e_{i}^{T}z_{t}^{*}\right)}^{2}=\max_{i\in I_{t}^{c}}\;{\left(z_{t}^{*}\right)}^{T}e_{i}e_{i}^{T}z_{t}^{*}\\ \; & \geq \frac{1}{R}{\left(z_{t}^{*}\right)}^{T}\sum_{i\in I_{t}^{c}}e_{i}e_{i}^{T}z_{t}^{*}\\ \; & =\frac{1}{R}{\left(z_{t}^{*}\right)}^{T}\;\left(\sum_{i\in I_{t}^{c}}e_{i}e_{i}^{T}+\sum_{i\in I_{t}}e_{i}e_{i}^{T}\;\right)\;z_{t}^{*}\\ \; & =\frac{1}{R}{∥z_{t}^{*}∥}^{2}, \end{array}$$

where the last but one line involves $z_{t}^{*}\in N\left(Q_{t}^{\left(1\right)}\right)$. Therefore,

(A20) $$L_{t}=e_{i_{t}^{*}}^{T}z_{t}^{*}\geq \frac{1}{\sqrt{R}}∥z_{t}^{*}∥.$$

At the same time, according to the Courant–Fischer theorem, along with the positive definiteness of $Q_{t}^{\left(2\right)}$,

(A21) $$L_{t}={\left(z_{t}^{*}\right)}^{T}Q_{t}^{\left(2\right)}z_{t}^{*}\leq \lambda_{\max}\left(Q_{t}^{\left(2\right)}\right)∥z_{t}^{*}{∥}^{2}<\ldots \left(Q_{t}^{\left(2\right)}\right){∥z_{t}^{*}∥}^{2},$$

where $\lambda_{\max}\left(Q_{t}^{\left(2\right)}\right)$ denotes the maximum eigenvalue of $Q_{t}^{\left(2\right)}$, and $\ldots (Q_{t}^{\left(2\right)})$ is the trace. Equations (A20) and (A21) together they give

(A22) $$∥z_{t}^{*}∥>\frac{1}{\sqrt{R}\ldots \left(Q_{t}^{\left(2\right)}\right)}=\frac{1}{\sqrt{R}\left(\frac{1}{t}\ldots \left(A_{t}^{\prime }\right)+N\beta_{t}^{\prime }\rho_{t}\right)}.$$

Substituting it back to (A20),

(A23) $$\max_{i\in I_{t}^{c}}e_{i}^{T}P_{t}^{-1}e_{i}\geq L_{t}\geq \frac{1}{\sqrt{R}}∥z_{t}^{*}∥>\frac{1}{R\left(\frac{1}{t}\ldots \left(A_{t}^{\prime }\right)+N\beta_{t}^{\prime }\rho_{t}\right)}.$$

Let $\rho^{*}=\frac{\delta }{NR}$, and $\rho_{t}^{*}=\rho^{*}-\frac{1}{t}\frac{\ldots (A_{t}^{\prime })}{N\beta_{t}^{\prime }}$. According to the sampling strategy Equation (A9), from previous results Equations (A10) and (A23), for t>T, $\rho_{t}<\rho_{t}^{*}$ implies $s_{t+1}\in I_{t}^{c}$. Define ${\tilde{\rho }}_{t}=\frac{1}{R}\sum_{i\in I_{t}^{c}}\eta_{i,t}$.

(A24) $$\begin{array}{cc} \rho_{t+1}\geq {\tilde{\rho }}_{t+1}=\frac{Rt{\tilde{\rho }}_{t}+1}{R(t+1)} & ={\tilde{\rho }}_{t}+\frac{1-R{\tilde{\rho }}_{t}}{R(t+1)}\\ \; & >{\tilde{\rho }}_{t}+\frac{(N-R)\delta }{R(t+1)}\\ \; & \geq \frac{\rho_{t}}{R}+\frac{(N-R)\delta }{R(t+1)}. \end{array}$$

By induction, this lower bound of $\rho_{t+\tau }$ increases with τ

(A25) $$\rho_{t+\tau }>\frac{\rho_{t}}{R}+\frac{(N-R)\delta }{R}\sum_{k=1}^{\tau }\frac{1}{t+k}$$

until $\rho_{t+\tau }\geq \rho_{t+\tau }^{*}$. Suppose that $\rho_{t-1}\geq \rho_{t-1}^{*}$ but $\rho_{t}<\rho_{t}^{*}$ at some t>T. Then,

(A26) $$\rho_{t}\geq \frac{t-1}{t}\rho_{t-1}\geq \frac{t-1}{t}\rho_{t-1}^{*}.$$

If $\lim_{t\to \infty }\frac{1}{t}\frac{\ldots (A_{t}^{\prime })}{N\beta_{t}^{\prime }}=0$, then $\lim_{t\to \infty }\rho_{t}^{*}=\rho^{*}$. We can obtain $\underset{t\to \infty }{lim inf}\rho_{t}\geq \lim_{t\to \infty }\frac{\left(t-1\right)\rho_{t-1}^{*}}{Rt}=\frac{\rho^{*}}{R}=\frac{\delta }{NR^{2}}>0$, which contradicts the definition. Therefore, the only possibility is R=0, and Lemma 1 is proved. □

The authors of Pronzato [28] have proved a similar statement in sequential DOE for nonlinear LS regression. In contrast, the proposed algorithm is an uncertainty sampling process for a Bayesian estimation problem, which makes our proposition and proof different in some way from theirs.

## Appendix D. Proof of Theorem 2

**Proof.** Let $U_{\epsilon }=\{f\in R^{N}\;|\;\forall \;i\in V,|f_{i}-f_{i}^{*}\left|<\epsilon \right\}$ be a “cubic” neighborhood around the true signal $f^{*}$, and its complement $U_{\epsilon }^{c}=R^{N}\setminus U_{\epsilon }$. We are to prove that $P(U_{\epsilon }^{c}|\Psi_{1:t},y_{1:t};\alpha_{t}^{\prime },\gamma_{t}^{\prime },$ $\beta_{t}^{\prime })$ converges to 0 with high probability for arbitrary ϵ>0. Here, for brevity, $\gamma_{t}^{\prime }$ is absorbed into $\alpha_{t}^{\prime }$. We can get a fractional form of the target

(A27) $$P\left(U_{\epsilon }^{c}|\Psi_{1:t},y_{1:t};\alpha_{t}^{\prime },\beta_{t}^{\prime }\right)=\frac{N_{t}}{D_{t}}=\frac{\int_{U_{\epsilon }^{c}}\frac{p(y_{1:t}|\Psi_{1:t},f;\beta_{t}^{\prime })}{p(y_{1:t}|\Psi_{1:t},f^{*};\beta^{*})}p\left(f;\alpha_{t}^{\prime }\right)df}{\int_{R^{N}}\frac{p(y_{1:t}|\Psi_{1:t},f;\beta_{t}^{\prime })}{p(y_{1:t}|\Psi_{1:t},f^{*};\beta^{*})}p\left(f;\alpha_{t}^{\prime }\right)df},$$

where $f^{*}$ is the true signal, and $\beta^{*}$ is the true noise precision. First look at the numerator. According to Corollary 2, as t→∞, $m_{i,t}\to \infty$. We have

(A28) $$\begin{array}{cc} \frac{p(y_{1:t}|\Psi_{1:t},f;\beta_{t}^{\prime })}{p(y_{1:t}|\Psi_{1:t},f^{*};\beta^{*})} & \overset{\left(a\right)}{=}\exp\;\left(-\sum_{i=1}^{N}m_{i,t}\;\left(\frac{1}{m_{i,t}}\sum_{j=1}^{m_{i,t}}\ln\frac{p(y_{i,j}|f_{i}^{*};\beta^{*})}{p(y_{i,j}|f_{i};\beta_{t}^{\prime })}\right)\;\right)\\ \; & \overset{\left(b\right)}{\to }\exp\;\left(-\sum_{i=1}^{N}m_{i,t}\;KL\left(p\left(y_{i}|f_{i}^{*};\beta^{*}\right)\;\left|\right|\;p\left(y_{i}|f_{i};\beta_{t}^{\prime }\right)\right)\right), \end{array}$$

where $y_{i,j}$ denotes the *j*-th observation of vertex *i*; KL(p||q) is the Kullback–Leibler divergence between two distributions; (a) takes into account the independence of different observations in y conditioned on Ψ, f, and β; and (b) obeys the Law of Large Numbers. For any $f\in U_{\epsilon }^{c}$, there exists k∈V, such that $|f_{k}-f_{k}^{*}|>\epsilon$. Regardless of the value of $\beta_{t}^{\prime }$,

(A29) $$\begin{array}{cc} KL\left(p\left(y_{k}|f_{k}^{*};\beta^{*}\right)\;\left|\right|\;p\left(y_{k}|f_{k};\beta_{t}^{\prime }\right)\right) & =\frac{1}{2}\;\left(\beta_{t}^{\prime }{\left(f_{k}-f_{k}^{*}\right)}^{2}+\frac{\beta_{t}^{\prime }}{\beta^{*}}-\ln\frac{\beta_{t}^{\prime }}{\beta^{*}}-1\right)\\ \; & >\frac{1}{2}\ln\left(1+\beta^{*}\epsilon^{2}\right). \end{array}$$

For i≠k, $KL\left(p\left(y_{i}|f_{i}^{*};\beta^{*}\right)\;\left|\right|\;p\left(y_{i}|f_{i};\beta_{t}^{\prime }\right)\right)\geq 0$ by definition. Moreover, from Corollary 1, there exists δ>0, such that $\frac{m_{k,t}}{t}>\delta$ for all large *t*. Thus, for $\epsilon_{1}=\frac{1}{2}\delta \ln\left(1+\beta^{*}\epsilon^{2}\right)>0$,

(A30) $$\begin{array}{cc} \frac{p(y_{1:t}|\Psi_{1:t},f;\beta_{t}^{\prime })}{p(y_{1:t}|\Psi_{1:t},f^{*};\beta^{*})}<\; & \exp\;\left(-\frac{1}{2}m_{k,t}\ln\left(1+\beta^{*}\epsilon^{2}\right)\right)\\ <\; & \exp(-t\epsilon_{1}) \end{array}$$

for all $f\in U_{\epsilon }$ for sufficiently large *t* with high probability. Therefore, when *t* becomes large enough, no matter what values $\alpha_{t}^{\prime }$ have, the numerator

(A31) $$N_{t}=\int_{U_{\epsilon }^{c}}\frac{p(y_{1:t}|\Psi_{1:t},f;\beta_{t}^{\prime })}{p(y_{1:t}|\Psi_{1:t},f^{*};\beta^{*})}p\left(f;\alpha_{t}^{\prime }\right)df<\exp\left(-t\epsilon_{1}\right)$$

with high probability. Then, come to the denominator. As $\alpha_{t}^{\prime }$, $\beta_{t}^{\prime }$ are estimated by MML,

(A32) $$D_{t}\geq {\tilde{D}}_{t}\left(\alpha ,\beta^{*}\right)=\int_{R^{N}}\frac{p(y_{1:t}|\Psi_{1:t},f;\beta^{*})}{p(y_{1:t}|\Psi_{1:t},f^{*};\beta^{*})}p\left(f;\alpha \right)df,$$

where α is allowed to be chosen arbitrarily. Consider arbitrary $\epsilon_{2}>0$. Analogous to above, as t→∞ and $m_{i,t}\to \infty$, we have

(A33) $$\begin{array}{cc} \exp\left(t\epsilon_{2}\right)\frac{p(y_{1:t}|\Psi_{1:t},f;\beta^{*})}{p(y_{1:t}|\Psi_{1:t},f^{*};\beta^{*})} & =\exp\;\left(\sum_{i=1}^{N}m_{i,t}\;\left(\epsilon_{2}-\frac{1}{m_{i,t}}\sum_{j=1}^{m_{i,t}}\ln\frac{p(y_{i,j}|f_{i}^{*};\beta^{*})}{p(y_{i,j}|f_{i};\beta^{*})}\right)\;\right)\\ \; & \to \exp\;\left(\sum_{i=1}^{N}m_{i,t}\left(\epsilon_{2}-KL\left(p\left(y_{i}|f_{i}^{*};\beta^{*}\right)\;\left|\right|\;p\left(y_{i}|f_{i};\beta^{*}\right)\right)\right)\;\right). \end{array}$$

Define $X_{\epsilon_{2}/2}=\left\{f\in R^{N}\;|\;\forall \;i\in V,KL\left(p\left(y_{i}|f_{i}^{*};\beta^{*}\right)\;\left|\right|\;p\left(y_{i}|f_{i};\beta^{*}\right)\right)<\frac{\epsilon_{2}}{2}\right\}$. For all $f\in X_{\epsilon_{2}/2}$, when *t* grows large enough,

(A34) $$\exp\left(t\epsilon_{2}\right)\frac{p(y_{1:t}|\Psi_{1:t},f;\beta^{*})}{p(y_{1:t}|\Psi_{1:t},f^{*};\beta^{*})}>\exp\;\left(t\frac{\epsilon_{2}}{2}\right)$$

with high probability. Note that the term inside integration is positive. We shrink the range of integration and obtain

(A35) $$\begin{array}{cc} \exp\left(t\epsilon_{2}\right){\tilde{D}}_{t}\left(\alpha ,\beta^{*}\right) & >\exp\left(t\epsilon_{2}\right)\int_{X_{\epsilon_{2}/2}}\frac{p(y_{1:t}|\Psi_{1:t},f;\beta^{*})}{p(y_{1:t}|\Psi_{1:t},f^{*};\beta^{*})}p\left(f;\alpha \right)df\\ \; & >\exp\;\left(t\frac{\epsilon_{2}}{2}\right)\int_{X_{\epsilon_{2}/2}}\;p\left(f;\alpha \right)df. \end{array}$$

We can always choose an α such that $\int_{X_{\epsilon_{2}/2}}\;p\left(f;\alpha \right)df>0$ since p(f;α) is Gaussian. Then, we get $\exp\left(t\epsilon_{2}\right){\tilde{D}}_{t}\left(\alpha ,\beta^{*}\right)\to \infty$ as t→∞. Therefore, the denominator

(A36) $$D_{t}\geq {\tilde{D}}_{t}\left(\alpha ,\beta^{*}\right)>\exp\left(-t\epsilon_{2}\right)$$

for all large *t* with high probability, where $\epsilon_{2}$ can be any positive number. Pick $0<\epsilon_{2}<\epsilon_{1}$. For $\epsilon_{3}=\epsilon_{1}-\epsilon_{2}>0$,

(A37) $$P\left(U_{\epsilon }^{c}|\Psi_{1:t},y_{1:t};\alpha_{t}^{\prime },\beta_{t}^{\prime }\right)=\frac{N_{t}}{D_{t}}<\exp\left(-t\epsilon_{3}\right)$$

for all sufficiently large *t* with high probability. We finally reach a conclusion that $P(U_{\epsilon }^{c}|\Psi_{1:t},y_{1:t};\alpha_{t}^{\prime },\beta_{t}^{\prime })$ converges to 0 with high probability as t→∞ for any positive ϵ. □

The proposed algorithm is more complex than classical empirical Bayes method discussed in Petrone et al. [27], because in our setting, hyperparameters exist not only in signal prior but also in observation model, and i.i.d. complete observations are substituted by partial ones selected by strategy. That is why a more complicated proof of consistent signal estimation in our algorithm is provided here.
